# Arts, mental distress, mental health functioning & life satisfaction: fixed-effects analyses of a nationally-representative panel study

**DOI:** 10.1186/s12889-019-8109-y

**Published:** 2020-02-11

**Authors:** Senhu Wang, Hei Wan Mak, Daisy Fancourt

**Affiliations:** 1 Department of Sociology, National University of Singapore, Singapore, Singapore; 2 Department of Behavioural Science and Health, University College London, 1-19 Torrington Place, London, WC1E 7HB UK

**Keywords:** Arts engagement, Mental health, Wellbeing, Fixed-effects, Longitudinal study

## Abstract

**Background:**

Arts engagement within communities is ubiquitous across cultures globally and previous research has suggested its benefits for mental health and wellbeing. However, it remains unclear whether these benefits are driven by arts engagement itself or by important confounders such as socio-economic status (SES), childhood arts engagement, previous mental health, personality, or self-selection bias. The aim of this study is to use fixed effects models that account for unidentified time-constant confounding measures to examine the longitudinal association between arts (frequency of both arts participation and cultural attendance), mental distress, mental health functioning and life satisfaction.

**Methods:**

Data from 23,660 individuals (with a mean age of 47 years) included in the UK Understanding Society wave 2 (2010–2012) and wave 5 (2013–2015) were analyzed. Aside from controlling for all time-constant variables using fixed-effects models, we additionally adjusted for time-varying demographic factors (e.g. age and marital status), health behaviors and social support variables.

**Results:**

After controlling for all time-constant variables and identified time-varying confounders, frequent arts participation and cultural attendance were associated with lower levels of mental distress and higher levels of life satisfaction, with arts participation additionally associated with better mental health functioning. Health-related and social time-varying factors were shown partly but not wholly to explain the observed associations.

**Conclusion:**

Arts engagement amongst the population as a whole may help enhance positive mental health and life satisfaction, and protect against mental distress. These results are independent of a wide range of time-constant confounding factors.

## Background

Arts engagement within communities is ubiquitous across cultures globally. In the UK alone, there are estimated to be over 40,000 choirs [1], 11,000 amateur orchestras [2], 50,000 amateur arts groups [2], 50,000 book clubs [3], 5000 amateur theatre societies [2], 3000 dance groups [2], 2500 museums [4], and 1300 theatres [5]. Previous research has shown that arts engagement has beneficial effects for both mental health and well-being [6–10]. It has been suggested that this could be due to multiple mechanisms including arts activities enhancing self-identity through the pursuit of skills, stimulation of creativity and self-expression [11], facilitating self-esteem and self-efficacy [12], building an individual’s social identity [13], reducing psychological and biological markers of stress [14]**,** providing cognitive stimulation [15, 16], enhancing social support [17, 18], reducing sedentary behaviours associated with depression [19], and supporting coping skills [20–22].

However, a challenge in studies that have been conducted is accounting for confounding factors that could in fact explain any association between arts engagement and both mental health and wellbeing, especially as many of these confounders include complex individual traits and previous life experiences that can be hard to capture fully. For example, arts engagement is socially patterned, with engagement in adult life related to broader social and cultural capital as well as education, income, engagement in childhood and the engagement of parents and wider peer groups [23, 24]. Similarly, arts engagement has been shown to vary based on childhood engagement and the engagement patterns of friends and family as an individual transitions to adulthood [25], and based on previous mental health [26]. Personality too has been shown to affect attitudes towards arts engagement [27]. As these factors are all themselves associated with mental health, it is possible that associations between arts and mental health could in fact be due to individual confounding factors [28, 29]. This is the case both for intervention studies, for which self-selection bias is likely to predispose individuals with higher levels of cultural capital, greater past experience, better previous mental health and open personality types to take part, and for analyses of observational data. Even longitudinal analyses observational data that adjust for such factors still may not entirely remove their effects [31].

Therefore, to address these issues we analyzed data from a nationally-representative and longitudinal cohort study and applied fixed effects (FE) regression models, which automatically adjust for all time-constant unobserved confounders and help reduce the risk of omitted variable bias, as well as additionally adjusting for identified time-varying confounders.

## Method

### Participants

This study used data from Understanding Society: The UK Household Longitudinal Study (UKHLS), which provides high-quality longitudinal panel data comprising a stratified and clustered General Population Sample of around 40,000 households. These analyses used data from wave 2 (2010–2012) and wave 5 (2013–2015) when questions on arts participation/cultural events were included. Of 54,554 respondents in wave 2, 37,389 were followed up in wave 5, and 25,051 of these (around 67%) responded to self-completion questionnaires on health and arts. After deleting 1391 cases with missing information (around 5%), the final analytic sample includes 23,660 respondents and 47,320 person-wave observations.

### Measures

Arts engagement was measured using 28 separate questions that were categorised into participation in active arts participation (“arts participation”) or attending cultural events (“cultural attendance”). *Arts participation* included dance (including ballet), singing to an audience or rehearsing for a performance (not karaoke), playing a musical instrument, writing music, rehearsing/performing in a play/drama, opera/operetta or musical theatre, taking part in a carnival/street arts event, learning or practising circus skills, painting, drawing, printmaking or sculpting, photography, film or video making as an artistic activity, using a computer to create original artworks or animation, taking part in textile crafts, wood crafts or any other crafts such as embroidery, knitting, reading for pleasure (not newspapers, magazines or comics), writing any stories, plays or poetry, or being a member of a book club where people meet up to discuss and share books.

*Cultural attendance* included attending a film at a cinema or other venue, an exhibition or collection of art, photography, sculpture or a craft exhibition, an event which included video or electronic art, an event connected with books or writing, street arts or a public art display or installation, a carnival or cultural specific festival, a circus, a play/drama, pantomime or musical, an opera/operetta, a classical music performance, a rock, pop or jazz performance, a ballet, a contemporary dance performance, or an African people’s dance or South Asian and Chinese dance. For each question, frequency of arts engagement was measured using five categories for participation in arts participation (never, once/twice per year, once per month, once per week, more than once per week) and four categories for attendance at cultural events (never, once/twice per year, once per month, once per week or more).

Given the well-known distinctions between mental health and multidimensional wellbeing [32], we explored three different outcome variables. *Mental distress* was measured with GHQ-12 (General Health Questionnaire); a well-validated scale derived from 12 items to measure the levels of respondents’ psychiatric illness. Items include depressive and anxiety symptoms, sleeping problems, and overall happiness [33]. UKHLS converts the answers to GHQ-12 questions to a single continuous scale ranging from 0 (the least distressed) to 12 (the most distressed), with a lower score indicating better mental health.

*Mental functioning* was measured using SF-12 (12-Item Short Form Health Survey), a widely used and reliable instrument that measures respondents’ general quality of life and focuses both on mental and physical health. It places a particular emphasis on the implications of any problems for ability to function as normal in everyday life [34]. The survey contains eight indicators formed of 12 items: physical functioning, role limitations due to physical health problems, bodily pain, general health, vitality, social functioning, role limitations due to emotional problems, and mental health [34]. UKHLS calculates the SF-12 Mental Component Summary (MCS) score by assigning higher weights to mental health related items (the latter six items). The MCS score ranges from 0 (the lowest mental functioning) to 100 (the highest mental functioning).

Subjective wellbeing comprises both affective aspects (such as happiness and pleasure in daily life and being free from negative affect) as well as cognitive-evaluative aspects (such as life satisfaction) [35]. We focused specifically on *life satisfaction*. This was measured using a single-item “overall, how satisfied are you with your life nowadays?” Responses range from 1 (completely unsatisfied) to 7 (completely satisfied) [36].

We used directed acyclic graphs to identify potential confounding factors that could influence both mental health and arts engagement [37]. As our statistical approach controlled automatically for any time-constant factors, even if unobserved (see ‘Statistics’), we restricted our identification of further confounders to those that vary over time. Identified demographic confounders included age, age squared, marital status (never married, married/cohabited, divorced/separated/widowed), presence of children in the household (no children, preschool children aged 0–4, primary school children aged 5–11, middle school children aged 12–15), employment status (inactive, unemployed, working class, intermediate class, service class), number of people in the household, logged household income, and data collection wave. In order to ascertain whether individuals who engaged in the arts simply led healthier lifestyles, which contributed to their mental health (perhaps as an underlying function of socio-economic status), we additionally controlled for a wide range of health behaviours which are often associated with mental health [38, 39]. These included self-reported sports activity ranking (from 0 ‘doing no sport at all’ to 10 ‘very active through sport’), smoking behaviour (current smoker, ever smoked, never smoked), drinking frequency in the last year (from 1 - never drink - to 8 - drink every day), and portions of fruits or vegetables eaten per day. We also adjusted for the extent to which health limits moderate activities to try and capture the health selectivity in participating in arts activities or events. Finally, in order to identify whether individuals who engaged in the arts simply had stronger social ties and more frequent social contact, we additionally controlled for family support and friend support measured using a 4-point scale from 1 (not at all/no family or friends) to 4 (a lot) for each of the following 3 questions: family/friends understand the way I feel; I can rely on family/friends; I can talk about my worries with family/friends. Principal component factor analyses were conducted to extract one factor for family support (eigenvalue = 2.35, variance explained = 78%, alpha = 0.86) and one factor for friend support (eigenvalue = 2.45, variance explained = 82%, alpha = 0.89). For more details about the distribution of variables, see Table 1.

**Table 1:** Descriptive statistics for all time-varying characteristics included in analyses as measured at baseline

Items	Variables (min. and max. values)	Whole sample %/ M (SD)	Arts participation		*p*-value	Cultural attendance		*p*-value
			Less than once per week %/M (SD)	Once per week or more %/ M (SD)		Less than once per month %/M (SD)	Once per month or more %/ M (SD)	
Arts participation	None	18.91						
	Once/twice last year	3.24						
	Once per month	8.09						
	Once per week	13.57						
	More than once per week	56.19						
Cultural attendance	None	20.86						
	Once/twice last year	24.82						
	Once per month	36.06						
	Once per week or more	18.25						
Mental health conditions	GHQ-12 mental distress (0–36)	11.16 (5.53)	11.35 (5.64)	11.00 (5.28)	<0.001	11.53 (5.74)	10.75 (5.07)	<0.001
	SF-12 mental functioning (0–100)	49.55 (9.74)	49.14 (10.08)	50.05 (9.29)	<0.001	49.29 (10.68)	50.19 (8.93)	<0.001
Demographic characteristics	Age (16–101)	47.19 (18.01)	46.93 (16.63)	49.59 (16.70)	<0.001	52.21 (16.76)	45.90 (16.16)	<0.001
	Age groups							
	< 30	15.92	18.42	14.83	<0.001	11.34	19.77	<0.001
	31–40	17.35	19.11	16.58		15.69	18.74	
	41–50	21.11	22.27	20.61		19.25	22.67	
	51–60	18.44	17.76	18.73		19.48	17.56	
	> 60	27.19	22.44	29.25		34.24	21.26	
	Marital status							
	Single/Never married	29.35	33.01	27.77	<0.001	25.30	32.76	<0.001
	Married/Cohabited	56.6	54.95	57.31		58.18	55.27	
	Separated/Divorced/Windowed	14.05	12.03	14.92		16.52	11.97	
	Presence of children							
	No children	67.96	63.24	70.00	<0.001	70.39	65.91	<0.001
	Preschool children 0–4	13.23	16.69	11.73		14.64	12.04	
	Primary school children 5–11	11.81	12.44	11.53		9.13	14.06	
	Middle school children 12–15	7.01	7.63	6.74		5.84	8.00	
	Employment status							
	Inactive	33.17	30.22	34.45	<0.001	41.35	26.30	<0.001
	Unemployed	4.55	5.67	4.06		5.63	3.64	
	Working class	19.44	26.11	16.55		21.56	17.65	
	Intermediate class	14.69	14.97	14.57		12.77	16.30	
	Service class	28.15	23.03	30.38		18.69	36.11	
	Number of people in household (1–16)	2.84 (1.39)	2.90 (1.35)	2.66 (1.26)	<0.001	2.65 (1.29)	2.81 (1.30)	<0.001
	Logged household income (0–10.5)	7.41 (0.68)	7.36 (0.66)	7.48 (0.68)	<0.001	7.30 (0.64)	7.60 (0.67)	<0.001
Health behaviors	Portions of fruits/vegetables eaten per day (0–11)	3.32 (1.57)	3.05 (1.52)	3.58 (1.57)	<0.001	3.21 (1.55)	3.60 (1.60)	<0.001
	Drinking frequency in the last year (1–8)	4.68 (1.96)	4.70 (1.95)	4.42 (1.87)	<0.001	4.78 (1.92)	4.27 (1.76)	<0.001
	Smoking behavior							
	Current smoker	7.91	23.24	16.28	<0.001	23.16	14.37	<0.001
	Ever smoked	17.73	35.42	39.21		37.61	38.44	
	Never smoked	74.36	41.34	44.51		39.22	47.19	
	Health limits moderate activities							
	Yes, limited a lot	18.39	9.96	7.02	<0.001	12.27	4.24	<0.001
	Yes, limited a little	38.06	18.23	17.51		21.48	14.57	
	No, not limited at all	43.55	71.81	75.47		66.25	81.19	
	Sporting frequency	3.49 (2.92)	3.11 (2.89)	3.57 (2.84)	<0.001	2.69 (2.83)	4.06 (2.80)	<0.001
Social support	Family support (−2.39–1.41)	0.00 (0.99)	−0.12 (1.03)	0.06 (0.94)	<0.001	−0.13 (1.08)	0.12 (0.94)	<0.001
	Friend support (−2.41–1.09)	0.00 (0.99)	−0.07 (1.01)	0.01 (0.95)	<0.001	−0.04 (0.99)	0.01 (0.93)	<0.001
Number of respondents		23,660						
Person-wave observations		47,320						

*Note.* GHQ-12 = 12-Item General Health Questionnaire. SF-12 MCS = 12-Item Short Form Health Survey Mental Component Summary. Proportions reported for categorical variables. Mean values reported for continuous variables. Standard deviations in parenthesis

### Statistics

Using Stata 14, we performed FE regression analyses; a sophisticated statistical technique commonly used in causal inference research. Compared with ordinary least square regression, which does not distinguish between within- and between-person variation, FE regression only focuses on within-person variation, examining how changes in frequencies of art engagement are linked to changes in mental health within each individual over time [40]. In doing this, FE regression eliminates the confounding effects of all time-constant variables (e.g. gender, ethnicity, social class, personality, previous arts engagement, previous mental health, previous education etc). As such, these factors cannot explain any association found. Further, FE regression considers time-varying confounders at both waves, not just at baseline, capturing their dynamic relationship with the exposure and outcome to better estimate the causal relationship.

We fitted nested models adding covariates stepwise. Model 1 automatically adjusted for time-constant variables. Model 2 controlled for time-varying demographic characteritics and wave. Model 3 additionally controlled for time-varying health behaviors and social support. However, as the factors in Model 3 could be seen to lie on the causal pathway (which would make adjusting for them inappropriate), Model 2 may present more appropriate estimates. We further assessed whether age and gender were moderators through including interaction terms.

Although the panel data only consist of two waves, the key variables in this study such as mental health and frequencies of art engagement had enough within variation (on average 35% of total variation is from within variation) allowing for accurate estimation of FE regression analysis (Allison, 2009).

Data were strongly balanced. A Hausman test confirmed the selection of a fixed effects over a random effects model. The modified Wald test for group-wise heteroscedasticity was significant so sandwich estimators were applied. Coefficients for all years were not jointly equal to zero, so time-fixed effects were included in the model. Longitudinal weights provided by the UKHLS were used to adjust for the complex survey design, non-response rate, unequal selection probabilities and non-random attrition across waves.

## Results

### Decriptive statistics

Table 1 reports decriptive statistics for the sample as a whole for all time-varying factors, and also compares descriptive characteristics amongst those of frequent and infrequent arts participation and cultural attendance. Overall, the sample had a mean age of 47.2 years (SD = 18.0, range = 16–101) and was 55.6% female.

At baseline, people who were frequently engaged in arts had lower levels of mental distress and higher levels of mental functioning and life satisfaction than those who were infrequently engaged as well as better health behaviors, more social support from family and friends, and higher socioeconomic status. Mental distress showed a negative correlation with mental functioning (r = − 0.73, *p* < .001) and life satisfaction (*r* = − 0.49, *p* < .001), while mental functioning and life satisfaction were positively correlated (0.47, *p* < .001).

### Mental distress

When adjusting for all identified confounders, significantly lower levels of mental distress were found amongst those who participated in arts activities more than once a week (coef. -0.29, 95% CI -0.56, − 0.01), or who attended cultural events once a week or more (coef. -0.42, 95% CI -0.76, − 0.08) (Table 2).

**Table 2:** Fixed effects models predicting the associations between frequencies of art engagement & mental health

	Model 1 Coefficients (CI)		Model 2 Coefficients (CI)		Model 3 Coefficients (CI)	
GHQ-12 Mental Distress						
Participation in arts activities (ref = None)						
Once/twice per year	−0.35	(−0.80, 0.11)	− 0.33	(− 0.78, 0.12)	−0.31	(− 0.75, 0.13)
Once per month	0.07	(− 0.29, 0.43)	0.04	(− 0.32, 0.39)	0.05	(− 0.30, 0.40)
Once per week	−0.19	(− 0.49, 0.11)	− 0.21	(− 0.51, 0.10)	− 0.21	(− 0.51, 0.09)
More than once per week	−0.31*	(− 0.60, − 0.03)	−0.34*	(− 0.62, − 0.05)	−0.29*	(− 0.56, − 0.01)
Within R-squared	0.00		0.01		0.03	
Attendance at cultural events (ref = None)						
Once/twice per year	−0.13	(− 0.40, 0.14)	− 0.14	(− 0.41, 0.13)	−0.14	(− 0.41, 0.12)
Once per month	−0.18	(− 0.46, 0.11)	− 0.18	(− 0.46, 0.11)	−0.14	(− 0.43, 0.14)
Once per week or more	−0.51**	(− 0.85, − 0.16)	−0.50**	(− 0.85, − 0.15)	−0.42*	(− 0.76, − 0.08)
Within R-squared	0.00		0.01		0.03	
SF-12 Mental Functioning						
Participation in arts activities (ref = None)						
Once/twice per year	0.71	(−0.02, 1.45)	0.56	(−0.17, 1.29)	0.51	(− 0.21, 1.23)
Once per month	0.13	(−0.47, 0.73)	0.11	(− 0.49, 0.70)	0.08	(− 0.51, 0.67)
Once per week	0.39	(−0.11, 0.90)	0.33	(−0.17, 0.83)	0.33	(−0.16, 0.82)
More than once per week	0.66**	(0.21, 1.12)	0.57*	(0.12, 1.02)	0.50*	(0.05, 0.95)
Within R-squared	0.00		0.02		0.03	
Attendance at cultural events (ref = None)						
Once/twice per year	0.28	(−0.18, 0.74)	0.30	(− 0.16, 0.76)	0.31	(− 0.15, 0.76)
Once per month	0.25	(−0.23, 0.73)	0.22	(−0.26, 0.71)	0.18	(−0.30, 0.65)
Once per week or more	0.63*	(0.05, 1.22)	0.56	(−0.03, 1.15)	0.49	(−0.09, 1.07)
Within R-squared	0.00		0.02		0.03	
Subjective wellbeing: Life satisfaction						
Participation in arts activities (ref = None)						
Once/twice per year	0.14*	(0.00, 0.28)	0.11	(−0.03, 0.25)	0.11	(−0.03, 0.25)
Once per month	0.08	(−0.02, 0.18)	0.06	(−0.04, 0.16)	0.06	(−0.04, 0.16)
Once per week	0.07	(−0.02, 0.16)	0.05	(−0.04, 0.14)	0.05	(−0.04, 0.14)
More than once per week	0.12**	(0.04, 0.20)	0.10*	(0.02, 0.18)	0.09*	(0.01, 0.17)
Within R-squared	0.00		0.02		0.03	
Attendance at cultural events (ref = None)						
Once/twice per year	0.14***	(0.06, 0.22)	0.13**	(0.05, 0.21)	0.13**	(0.05, 0.21)
Once per month	0.14**	(0.06, 0.23)	0.12**	(0.04, 0.21)	0.11*	(0.03, 0.20)
Once per week or more	0.21***	(0.11, 0.32)	0.19***	(0.08, 0.29)	0.17**	(0.07, 0.27)
Within R-squared	0.00		0.02		0.03	

Note: Observations: 47,320. Respondents: 23,660. GHQ-12 = 12-Item General Health Questionnaire. SF-12 MCS = 12-Item Short Form Health Survey. Model 1 = automatically adjusted for time-constant variables. Model 2 additionally adjusted for demographic factors (age, age squared, marital status, presence of children, employment status, number of people in household, household income and wave). Model 3 additionally adjusted for health-related and social factors (extent to which health limits moderate activities, portions of fruits or vegetables eaten per day, smoking behavior, drinking frequency, sporting frequency, family support and friend support). Confidence intervals in parentheses. *** *p* < 0.001, ** *p* < 0.01, * *p* < 0.05

### Mental functioning

When accounting just for time-constant factors, participation in arts activities was associated with significantly higher mental functioning (coef. 0.66, 95% CI 0.21, 1.22), with this result maintained when considering all identified time-varying confounders (coef. 0.50, 95% CI 0.05, 0.95) (Table 2). The association between attending cultural events and mental functioning shown when just accounting for time-constant factors (coef. 0.63, 95% CI 0.05, 1.22) was attenuated when controlling for time-varying confounders.

### Subjective wellbeing: life satisfaction

When adjusting for all identified confounders, people who participated in arts activities more than once a week (coef. 0.09, 95% CI 0.01, 0.17), or who attended cultural events at least once/twice per year (coef. 0.13, 95% CI 0.05, 0.21) had significantly higher life satisfaction than those who had not participated in these art activities or cultural events (Table 2).

## Discussion

Our study showed a relationship between both frequent arts participation and cultural attendance and lower levels of mental distress and life satisfaction. The results were particularly strong for life satisfaction, for which there were also associations from less frequent cultural attendance. Arts participation was also associated with higher levels of mental functioning. Our results therefore confirm a relationship between arts engagement and multiple different aspects of mental health. This supports previous work using representative samples that had similar findings [41–44]. But this finding builds on these previous studies by using a sophisticated statistical technique that shows that although arts engagement is associated with broader aspects of social and cultural capital and socio-economic status (which are themselves associated with health) [28], the relationship is independent of these factors. This finding goes against a finding from a recent study using Swiss data that specifically explored the role of socio-economic status as a confounder, but is supported by another recent study exploring the same question using UK data of older adults [41, 45]. Further, our analyses automatically accounted for other time-constant factors such as personality, previous arts engagement, and previous mental health, suggesting that the associations found in this study could not be explained by any of these factors.

This study is observational and limited by only having data across two waves of UKHLS which do not permit lagged analyses. Therefore, this paper does not attempt to show the direction of the relationship. Instead, it focuses on showing the independence of this relationship from time-constant factors. However, there is a large literature using experimental approaches showing that arts activities can affect mental health [10]. Arts engagement can be considered as a ‘complex’ or ‘multi-modal’ health-promoting activity in that it combines multiple health-promoting or risk-reducing factors such as gentle physical activity, social interaction, relaxation, emotional expression, and cognitive stimulation [10, 46]. Our analyses could shed further light onto the causal mechanisms that could link arts and mental health as we found a relationship for higher mental health functioning only with arts participation; not cultural attendance. As cultural attendance is associated with improved mental distress and life satisfaction directly but not *functioning*, this suggests a direct relationship with affective symptoms (such as reductions in negative feelings and stress hormones and enhanced feelings of happiness) but not with an ability to alter psychological or behavioural factors relating to coping with affective symptoms. However, arts participation does show a relationship with mental functioning. As the major distinction between the two types of arts engagement is the participation itself, as multiple other elements of the two types of activities (e.g. aesthetic engagement, gentle physical activity, social interaction etc) are consistent, this suggests it is *participation* that supports coping. Whether there are different causal effects of arts participation and cultural attendance on mental functioning remains to be explored further. But this proposed difference in mechanisms between the two types of engagement is supported by several intervention studies that have identified improvements in aspects of functioning such as self-efficacy, agency and purpose as a result of arts participation [47].

Our study has a number of strengths including its large, representative sample, its longitudinal design, its use of a statistical model that considers both time-constant and time-varying factors, its rich data on different types of arts engagement, and its comparison of multiple related measures of mental health and wellbeing. However, it is possible that residual confounding for time-varying factors remains. Nevertheless, as all time-constant factors are automatically considered and the data allowed us to include all identified confounders, remaining unobserved heterogeneity should be small. Our analyses also focused on broad categories of arts engagement. Future research may like to consider different types of arts or cultural activities in greater detail and consider how access to activities within communities may affect abilities to engage. Finally, given that the data used in this study was from a national, representative data set and that a large sample size was maintained for the analyses, the generalizability is assumed to be relatively high. However, further work is required to examine whether the findings of this study can be replicated in different settings (such as in data from countries or more specific samples), and whether this finding is common sub-groups, in particular individuals with mental illness.

## Conclusions

Overall, our results demonstrate associations between arts participation, cultural attendance and multiple aspects of mental health and wellbeing. Importantly, we found that such associations are independent of time-constant socio-demographic, historical or personality-based factors. Future large-scale studies are encouraged to explore the potential of arts interventions as a public health strategy to help promote both mental health and wellbeing.

## Data Availability

UKHLS data is available from the UK Data Service https://discover.ukdataservice.ac.uk/catalogue/?sn=6614. Data documentation is available from the Understanding Society website https://www.understandingsociety.ac.uk/documentation.

## Abbreviations

FE: Fixed effects
GHQ-12: General Health Questionnaire
MCS: Mental Component Summary
SF-12: 12-Item Short Form Health Survey
UKHLS: UK Household Longitudinal Study
